# Ultrafast-light driven plasmonic inactivation of *S. epidermidis*: mechanistic insights

**DOI:** 10.1039/d5tb02670a

**Published:** 2026-07-13

**Authors:** Ramprasath Rajagopal, Koustav Kundu, Saatwik Suman, Ainsley Gray, Lawrence D. Ziegler, Shyamsunder Erramilli, Björn M. Reinhard

**Affiliations:** a Departments of Physics, Boston University Boston Massachusetts USA; b Departments of Chemistry, Boston University Boston Massachusetts USA bmr@bu.edu; c The Photonics Center, Boston University Boston Massachusetts USA

## Abstract

Biofilm infections and antimicrobial resistance are growing problems that necessitate alternative strategies to supplement or replace antibiotic drugs. Plasmonic antimicrobials utilize localized thermal, chemical and photophysical responses to the optical excitation of localized plasmons in noble metal nanostructures for microbial inactivation. High fluence femtosecond pulsed laser excitation of noble metal nanoparticles allows for highly non-linear physics and gives rise to a wide range of physico-chemical phenomena, such as plasma, reactive oxygen species generation, and nanocavitation, which also have antibacterial implications. In this work, we irradiate lipid functionalized gold nanorods that have a longitudinal plasmon resonance at 710 nm bound to Gram-positive *Staphylococcus epidermidis* with 5–9 mJ cm^−2^ 85 fs pulses centered at 812 nm and demonstrate a bacterial inactivation of up to 50%. We show that this inactivation is driven by ROS generation and possibly cell permeabilization through shockwaves. The moderate fluence and off-resonant nanorod excitation is found to provide a tradeoff between ROS generation and minimizing nanorod deformation, laying the foundation for future multi-modal plasmonic therapies and a more complete understanding of their antimicrobial mechanisms.

## Abstract 2

The inactivation of *Staphylococcus epidermidis* through gold nanorods under ultrafast-light irradiation is investigated.

## Graphical abstract

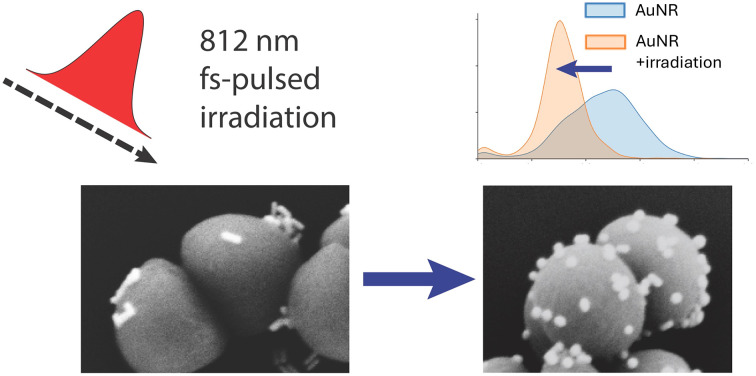

## I. Introduction

Antimicrobial resistance (AMR) is a global public health issue that is projected to grow rapidly, resulting in 10 million deaths annually by 2050.^1^ Several biological,^2,3^ environmental,^4^ and socioeconomic factors are involved in the growth of AMR and its human impact. As part of an arsenal for tackling this problem, the development of new antimicrobial treatments and a better understanding of antimicrobial mechanisms are imperative.^1^

Plasmonic antimicrobials^5^ utilize the large optical cross-sections of noble metal nanoparticles to generate heat and catalyze the formation of reactive oxygen species (ROS),^6–10^ that can inactivate microbial targets. Plasmon based systems have previously been used to inactivate bacteria in different configurations, including as photothermal agents^11–15^ or in combination with photosensitizers under continuous wave or nanosecond pulsed excitation.^16–19^ Of particular note are prior applications that demonstrated an antibacterial effect against biofilms in wound models.^20,21^ Plasmonic nanomaterials in combination with ultrafast light provide additional opportunities to generate high power densities on the nanoscale that can give access to additional phenomena. For instance, after conditions are met for nanocavitation, shock waves can be launched that can inactivate microbes.^22,23^ However, at the necessary high power densities, restructuring of plasmonic nanoparticles can lead to time-dependent changes in heat, ROS generation,^10^ bubble and shockwave generation.^24–26^ In this work, we therefore set out to investigate key mechanistic effects of bacterial inactivation by bound nanoparticle and ultrafast light strategies, such as the role of ROS generation and the structural reshaping of the nanoparticles. We performed this work with *Staphylococcus epidermidis* (*S. epidermidis*), a commensal Gram-positive coccus bacteria commonly found in human epithelia, but which can lead to nosocomial infections, most commonly through biofilms formed on medical instruments.^27^ Such infections can develop resistance to host defenses and antibiotics. We previously demonstrated ROS generation by gold nanorods (AuNR) under excitation with ultrafast light. These studies also revealed that a prolonged effect required adjustment of the fluence to counterbalance ROS generation efficiency and structural deformation of the nanorods.^10^ To translate this insight towards bacterial inactivation, we choose a well-studied, non-virulent, and non-biofilm forming variant of *S. epidermidis* (ATCC 12228) and targeted them with lipid wrapped AuNR. The lipid composition includes 1,2-dioleoyl-snglycero-3-ethylphosphocholine (EPC) for a positive surface potential that facilitates binding of the nanoparticles to the negatively charged bacterial surface. The bacteria were then treated with ultrafast light centered at 812 nm for 30 min at fluences up to 9 mJ cm^−2^ in the presence of AuNR and gold nanospheres (AuNS) exhibiting different degrees of overlap with the excitation wavelength.

We detected significant levels of inactivation of *S. epidermidis* targeted by AuNR with detuned longitudinal resonance and show that ultrafast light induced ROS formation is a key factor for the observed bacterial inactivation. The potential impact of photothermal effects is examined with a computational model. Intriguingly, AuNR and AuNS were found to bind to bacteria with reduced aggregation after irradiation with ultrafast light than before. These processes provide a mechanistic framework for understanding the different contributions to bacterial inactivation achieved with femtosecond irradiation of AuNR.^5,23,28,29^ The potential of ultrafast excitation of AuNR for future antimicrobial therapies is discussed.

## II. Methods

### II.A. Materials

Hexadecyltrimethylammonium bromide (Sigma-Aldrich, ≥98%, H5882-500G), gold(iii) chloride trihydrate (≥99.9% trace metals basis, 520918-1G), silver nitrate (ACS reagent, ≥99.0%, 209139-25G), sodium borohydride (Sigma-Aldrich, 99%, 213462-25g), l-ascorbic acid (ACS reagent, ≥99%, 255564-100G), hydrochloric acid (ACS reagent, 37%, 258148-100 ML), 1-octadecanethiol (purum, ≥95.0%, 74731-50G), Mn(iii)tetrakis(4-benzoic acid)porphyrin chloride (MnTBAP) (>95.0%, 475870-25MG), d-mannitol (>98%, M4125-100G), Sodium Azide (≥99.5%, S2002-25G), and water (ACS reagent, 320072-2.5L) were purchased from Sigma-Aldrich. Sodium oleate (97%, O0057) was purchased from TCI America. 1,2-Dipalmitoyl-*sn-glycero*-3-phosphocholine (DPPC, 850355), cholesterol (700100), 1,2-dioleoyl-*sn-glycero*-3-ethylphosphocholine (chloride salt) (18:1 EPC, 890704) and 1,2-dioleoyl-*sn-glycero*-3-phospho-l-serine (sodium salt) (18:1 DOPS, 840035) were purchased from Avanti Polar Lipids. All glassware and stir bars were cleaned with aqua regia and piranha before using for the synthesis of nanoparticles and liposomes, respectively. The Invitrogen LIVE/DEAD® BacLight™ Bacterial Viability Kit (L13152) was purchased from Thermofisher Scientific.

### II.B. Nanoparticle synthesis

#### II.B.1. Synthesis of AuNR_710 and AuNR_750

Synthesis of the AuNR were carried out following an established protocol by Murray *et al.* with slight modifications.^30^ The seed solution was prepared by mixing 5 mL of 0.2 M CTAB and 5 mL of 0.5 mM HAuCl_4_·3H_2_O under mild stirring at 30 °C. Next, 0.6 mL of ice cold NaBH_4_ of 10 mM was added to it at 1200 rpm. The solution changed its color from yellow to brown immediately and stirred for another 2 minutes. The seed solution was then incubated for 30 minutes at 30 °C before use.

For the growth solution 2.4 mL of 4 mM AgNO_3_ was added to a 25 mL aqueous solution containing 37 mM CTAB and 76 mM sodium oleate and incubated for 15 minutes at room temperature. Next, 25 mL of 1 mM HAuCl_4_·3H_2_O was added to the solution at a stirring speed of 700 rpm. The solution was stirred for 90 minutes during which the color changes from yellow to colorless. Next, 0.2 mL and 0.25 mL of 37% HCl were added to it for AuNR_710 and AuNR_750 respectively, followed by the addition of 0.125 mL of ascorbic acid of 64 mM. The solution was stirred at 400 rpm for 15 minutes. Lastly, 0.125 mL of seed solution was added at 1200 rpm and the solution was stirred for 30 s, followed by incubating overnight at 30 °C. The AuNRs were collected by centrifugation at 5000 rpm and washed twice with water before further use.

#### II.B.2. Synthesis of AuNS

The synthesis of AuNS was done following an adapted Turkevich method.^31^ First 20 nm AuNS were prepared by reducing 20 mL of 0.254 mM HAuCl_4_·3H_2_O at 110 °C with 0.8 mL of 34 mM sodium citrate dihydrate. Next, it was used as a seed solution to grow 60 nm AuNS. 20 mL of this seed solution was diluted to 100 mL with water, followed by simultaneously adding 80 mL of 0.9 mM HAuCl_4_·3H_2_O and 80 mL solution containing 0.85 mM sodium citrate dihydrate and 2.84 mM ascorbic acid, dropwise at 400 rpm at room temperature.

#### II.B.3. Lipid coating of AuNR and AuNS

The lipid coating on both AuNR and AuNS was formed following an established protocol.^10^ For positively charged lipid-coated nanoparticles, a mixture of DPPC (50 mol%), cholesterol (40 mol%) and EPC (10 mol%) of total 2 µmoles in chloroform was rotary evaporated to make a lipid film in a round bottom flask. Next, it was desiccated for 6 hours, and the film was rehydrated with 20 mM HEPES keeping the final lipid concentration at 1 mM. The liposomes were made by probe sonication for 5 minutes. Next, 0.16 mL of 1 octadecanethiol of 2 mg mL^−1^ in ethanol was added to it, followed by adding 1 mL of AuNR or AuNS solution containing approximately 10^10^ particles. The solution was incubated overnight to generate lipid-coated nanoparticles. The particles were washed twice by centrifugation to remove excess lipids at 5000 rpm before use.

For the negatively charged lipid-coated nanoparticles, 10 mol% EPC was replaced by 0 mol% DOPS in the lipid mixture.

### II.C. Optical setup

A modified Coherent Elite Duo system provided femtosecond pulses centered at 812 nm at a 1 kHz repetition rate. The full width at half maximum (fwhm) pulse width is 85 fs, measured through autocorrelation. The fwhm beam width measured with a knife-edge test, fitted to a Gaussian beam, is 0.48 cm, and the spectral width is 22.9 nm. The collimated femtosecond linearly polarized NIR beam was passed through a 7 mm diameter iris before passing through the quartz cuvette. 9 mJ cm^−2^ is the unattenuated beam's maximum fluence measured just before the sample, lower values were achieved through addition of a variable attenuator. All given fluence values in the rest of the manuscript correspond to the fluence measured at the center of the beam.

### II.D. Bacterial growth, treatment, and quantification

*S. epidermidis* (ATCC 12228) was grown 16–20 h in a low-salt variant of Lysogeny Broth (LB) Media containing 10 mM NaCl. The overnight culture (OD 2–3) was centrifuged at 1000 RCFs for 5 min, and resuspended in 20 mM HEPES and 10 mM NaCl (ls-HEPES) buffer.

Bacterial samples were diluted with appropriate amounts of gold nanoparticles in ls-HEPES buffer such that the final volume was 1.5 mL and bacterial OD was 0.2. Before exposures, we ensured stirring (∼150 rpm) of 5 min.

This bacterial-nanoparticle samples were held in quartz cuvettes under stirring (∼150 rpm) within a temperature-controlled environment to maintain the mixture at 20 °C. They were then exposed to ultrafast pulses with fluences up to 9 mJ cm^−2^ for 30 min.

To quantify bacterial inactivation, serial dilution and plate assays on LB agar plates were performed before and after laser exposure to account for any aggregation of the nanoparticle-bacterial mixtures.

#### II.D.1. ROS scavengers

To test the effect of ROS on inactivation, ROS scavenger solutions in 20 mM HEPES buffer were prepared and added to samples. The scavengers and their final concentration in samples were: 10 mM NaN_3_ (singlet oxygen radical scavenger), 10 mM Mannitol (hydroxyl radical scavenger), and 0.02 µM MnTBAP (superoxide anion radical scavenger)

## III. Results and discussion

### III.A. Binding of nanoparticles

**Fig. 1:**
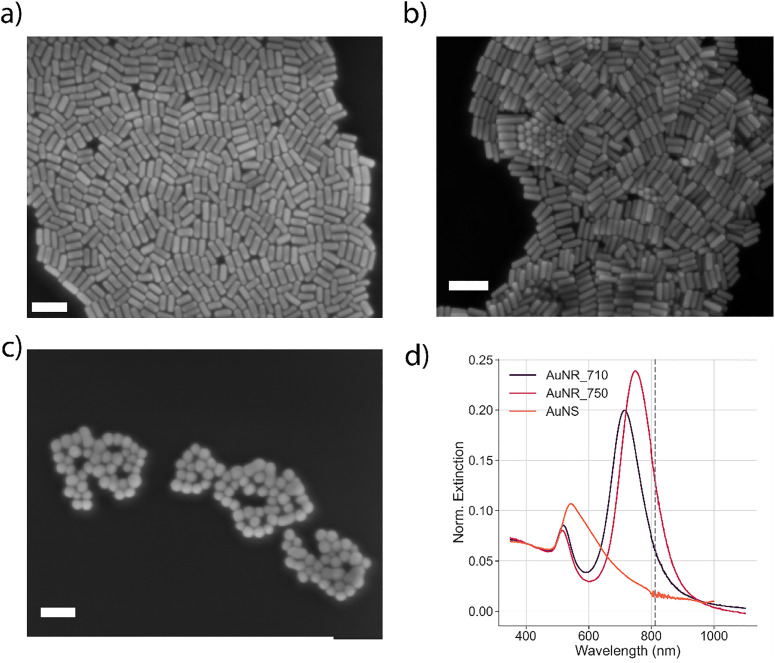
SEM images of (a) AuNR_710, (b) AuNR_750, and (c) AuNS. Scale bars are 200 nm. (d) UV-vis spectra of lipid wrapped AuNS, AuNR_750, and AuNR_710 under the conditions of the bacterial inactivation studies. AuNS and AuNR_750 spectra are normalized to match 400 nm extinction of AuNR_710.

In this proof-of-concept study, binding interactions between positively charged, lipid-coated nanoparticles and *S. epidermidis* were utilized to explore the antibacterial effect of ultrafast light excited noble metal nanoparticles bound to bacteria and the underlying mechanisms. Positively charged lipid wrapped gold nanoparticles were obtained by inclusion of EPC in the lipid membrane, while negatively charged nanoparticle controls contained DOPS. SEM images and UV-Vis spectra of the AuNR and AuNS used in this work are provided in Fig. 1. Structural parameters, as well as absorption and scattering cross sections at 812 nm are summarized in the SI (Table S1). The AuNS plasmon resonance exhibits negligible spectral overlap with the excitation wavelength, whereas the two AuNR show different degrees of overlap. AuNR_750 with aspect ratio 2.9, whose longitudinal plasmon resonance peaks at 750 nm, exhibit more overlap than AuNR_710 with an aspect ratio of 2.5 and a peak plasmon resonance of 710 nm (Fig. 1d).

AuNR_710 were incubated with bacteria in ls-HEPES buffer pH 7 (1.5 mL) in a ratio of 40 : 1. This ratio was chosen to avoid the formation of large bacterial and nanoparticle aggregates, and to minimize the red-shift and broadening of the AuNRs’ longitudinal resonance. We leave a detailed investigation of the effect of the particle to bacteria ratio for a future study. For other nanoparticle preparations, the OD 400 nm (∼0.06–0.07) was matched to that of AuNR_710 to maintain a constant amount of gold between different preparations.^10^ Due to the AuNPs’ similar particle volumes (see SI Table S1), matching OD 400 nm also matches particle concentrations and particle to bacteria ratios to within ∼20% across AuNPs. The LB growth media and ls-HEPES buffer were chosen to have 10 mM NaCl to facilitate bacterial growth and electrostatic interactions during the binding experiments. Bacteria were incubated with nanoparticles in an aqua-regia cleaned quartz cuvette with stirbar and stirred for at least 5 min. Then, samples were irradiated with varying intensities of femtosecond 812 nm irradiation for 30 min under stirring while the sample cuvette was maintained at 20 °C through use of a temperature controller.

**Fig. 2:**
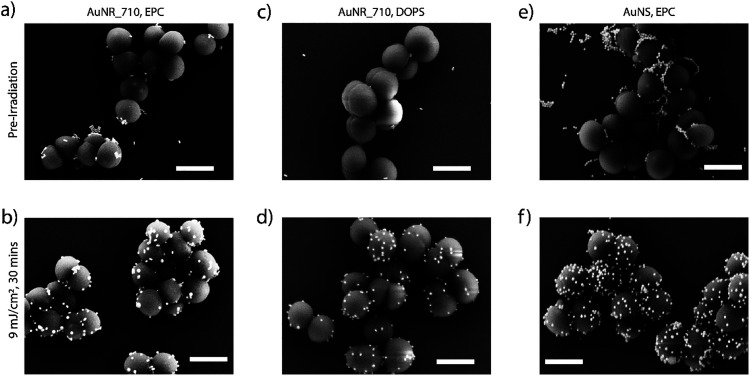
SEM images of various AuNP preparations incubated with bacteria taken before and after irradiation at 9 mJ cm^−2^ for 30 min: EPC AuNR_710 before (a) and after (b) irradiation, DOPS AuNR_710 before (c) and after (d) irradiation, and EPC AuNS before (e) and after (f) irradiation. The aggregation of binding nanoparticles before irradiation onto bacterial surface induces a red-shift of the longitudinal resonance. Post irradiation all conditions show binding to bacteria with greater spatial separation between the particles. Scalebars are 1 µm.

**Fig. 3:**
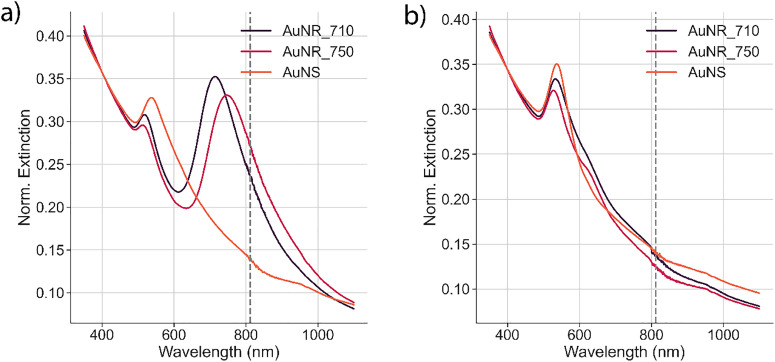
(a) UV-Vis spectra of various lipid wrapped EPC AuNP incubated with *S. epidermidis* before laser irradiation. (b) UV-Vis spectra of EPC AuNP and *S. epidermidis* samples after irradiation.

We observed binding of positively charged AuNR_710 (Fig. 2a) and AuNS (Fig. 2e) to *S. epidermidis*, but not of negatively charged AuNR_710 (Fig. 2c). For these binding studies, we chose to focus on AuNR_710 over AuNR_750, due to overall higher inactivation results, discussed in a latter section, and tighter distributions of various nanorod properties (see SI). The positively charged nanoparticles tend to cluster on the bacterial surface, leading to a broadening and red-shift of the plasmon resonance (Fig. 3a). Intriguingly, after irradiation at 9 mJ cm^−2^, deformed AuNR and AuNS, independent of the initial surface charge, exhibited an efficient and well-dispersed binding to *S. epidermidis* (Fig. 2(b, d and f)). This binding occurs despite a negative *ζ*-potential of −20 to −25 mV for all nanoparticles after irradiation. We comment on this in a latter section.

### III.B. Deformation of nanoparticles

The fluences used in this study are sufficiently high to induce a deformation of AuNR into increasingly spherical shapes after irradiation, as shown in Fig. 2(b, d and f).^10^ A notable effect of these structural changes is a decrease in the constraints of the nanoparticle diffusion in the vicinity of the bacteria. Nanorods have a reduced rotational diffusion coefficient close to the bacterial surface,^32^ but this effect weakens with decreasing aspect ratio. We also find that the bacterial rotational diffusion coefficient, estimated from the Stokes–Einstein–Debye relation (eqn (1)), is about 1000× smaller as compared to that of free AuNS or the volume equivalent sphere of AuNR.^32^1
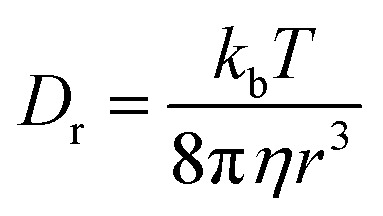
Hence, a longer time is needed for bound nanorods initially oriented away from the irradiation polarization to be deformed, than in the case of free nanorods.

### III.C. Impact of nanoparticle morphology

**Fig. 4:**
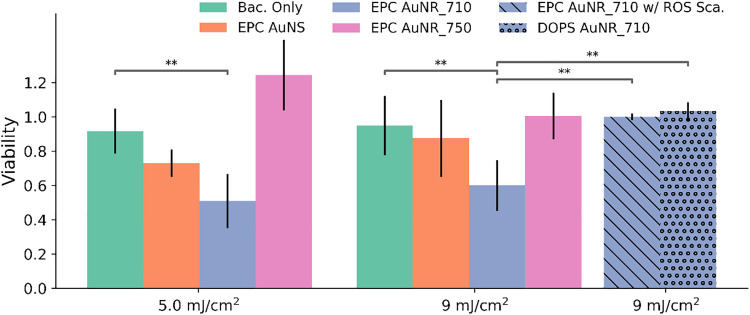
Viability of *S. epidermidis* determined by plate counts upon incubation and irradiation with various lipid wrapped AuNPs containing EPC or DOPS. There is a significant reduction in viability of AuNR_710 subject to either 5 or 9 mJ cm^−2^ irradiation for 30 min. When AuNR_710 is coated with DOPS, there is a significant recovery in viability as compared to AuNR_710 coated with EPC. Likewise, addition of ROS scavengers inhibits bacterial inactivation. At least 2 biological replicates were performed for each condition, error bars denote standard deviations, and statistical tests were done with 1-tail *T*-tests, ** denotes *p* < 0.05. 3 biological replicates were performed for ‘Bac. Only’@5 mJ cm^−2^, EPC AuNR_710@5, 9 mJ cm^−2^, DOPS AuNR_710@9 mJ cm^−2^, EPC AuNR_750@5 mJ cm^−2^. 4 biological replicates were performed for ‘Bac. Only’@9 mJ cm^−2^.

**Fig. 5:**
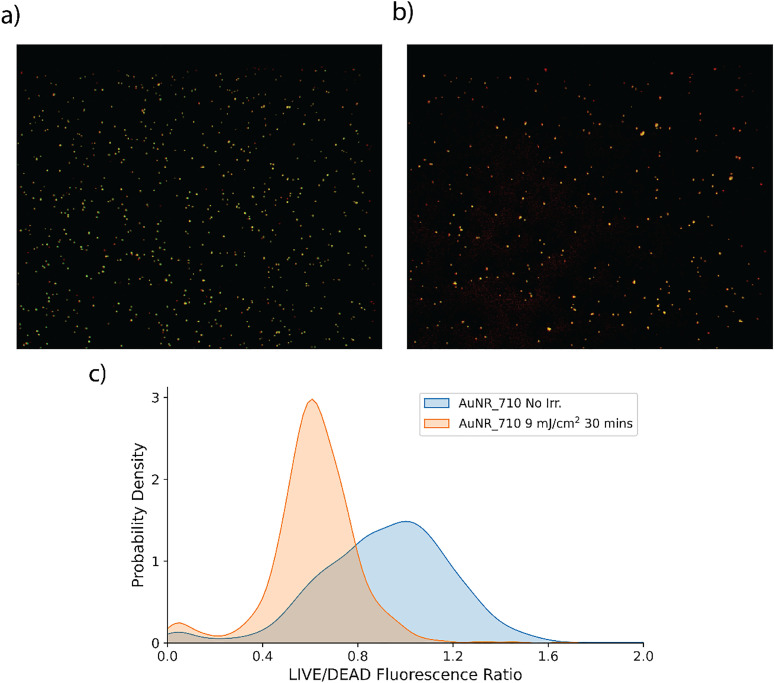
Fluorescence live-dead assay of *S. epidermidis* incubated with AuNR_710 before (a) and after (b) irradiation at 9 mJ cm^−2^ for 30 min. Images are gamma-adjusted for better visualization. Red denotes the membrane impermeable counter-stain PI fluorescence whereas green denotes fluorescence from the membrane permeable STYO 9 nuclear stain. Note the increased red fluorescence post-treatment (c) Kernel density estimate plots of LIVE/DEAD fluorescence ratios (green/red) of 2000 (post-irradiation, red) and 3800 (pre-irradiation, blue) individual bacterial clusters collated from 2 independent experiments and 4 images for each condition.

We tested AuNR with different aspect ratios and AuNS to evaluate the morphology dependence of the bacterial inactivation. Significant bacterial inactivation was detected for AuNR_710, but not for AuNR_750 or AuNS (Fig. 4) at both 5 mJ cm^−2^ and 9 mJ cm^−2^. Live-dead staining using a combination of membrane permeable SYTO-9 and membrane impermeable Propidium Iodide (PI) dyes (Fig. 5) indicates membrane damage and/or cell death induced by the irradiated AuNR_710 when bound to the bacterial cells.^33^ The estimated probability density distributions in Fig. 5c, constructed from the fluorescence ratio, SYTO-9 emission divided by PI emission, of individual bacterial clusters, show a clear uniform shift post-treatment to a lowered fluorescence ratio, whereas viability studies in Fig. 4 indicate a reduction by about 50%. The presence of AuNPs did not noticeably affect the fluorescence ratio (see SI). We conclude from these observations that most cells experience greater cell permeability but only a proportion experience cell death, presumably due to bacterial membrane repair mechanisms.

We attribute the stronger antibacterial effect for AuNR_710 relative to AuNR_750 nanoparticles, to differences in the spectral overlap with the excitation laser. For both types of rods UV-Vis spectra indicate a reshaping into spheres under the conditions of the experiment (Fig. 3). But due to the stronger overlap with the excitation laser, the structural transformation will be faster for AuNR_750. Consequently, AuNR_710 can provide a plasmonic response for a longer time than AuNR_750. AuNS are off-resonant under all conditions and provide a weak plasmonic response at the excitation wavelength of 812 nm. We note that negatively charged DOPS-coated AuNR_710 did not exhibit any reduction in viability (Fig. 4), which indicates that initial binding of AuNR to the bacteria prior to ultrafast laser irradiation (after which all nanoparticles were observed to bind) *is required* for the observed antibacterial effect.

### III.D. Mechanism of inactivation

Under the chosen experimental conditions, ultrafast light excitation of AuNR, can result in ROS production, photothermal effects, as well as bubbles and shockwave generation. We assess the extent of the photothermal effects of the AuNR through the Two-Temperature Model (TTM) with a cylindrical heat equation in the bulk.^34,35^ For details of the model, see (SI). We consider the heating effects at incident pulse peak fluence and for the case of AuNR_710 aligned with their long axis parallel to the laser polarization direction. We characterize the threshold fluence just before the onset of the bulk photothermal bubbling threshold of 550 K, 1 nm away from the nanorod surface. Due to optical non-linearities^36^ and the possibility of plasma mediated cavitation,^37^ this threshold fluence is likely an under-estimate. For AuNR_710, the bulk photothermal bubbling threshold occurs near ∼5.6 mJ cm^−2^. The corresponding threshold for AuNR_750 occurs near ∼1.5 mJ cm^−2^ (see SI).

**Fig. 6:**
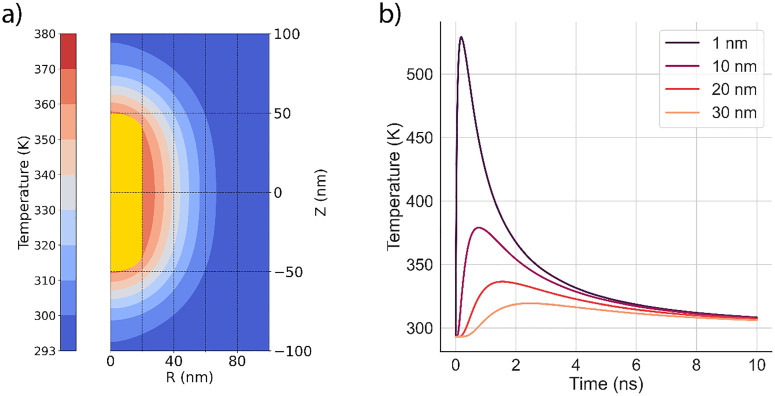
(a) Contour plot of temperature of water surrounding AuNR_710, 2 ns after irradiation with a pulse energy just below the bubbling threshold. (b) Corresponding temperature (*K*) *vs.* time at various radial distances from the waist of the gold AuNR (*Z* = 0). 1 nm corresponds to the typical length used for determination of the photothermal bubbling threshold.

Based on this model, the photothermal effect could play a role up to 30 nm from the nanorod surface with a maximum temperature of 320 K as seen in Fig. 6. Estimates of the Staphylococcal cell wall thickness (from *S. Aureus*) range between 20–40 nm.^38^ Given the estimated ∼few nm thickness of the lipid wrapped layer on the AuNR, we expect that the damage from photothermal effects is thus most likely constrained to within the cell wall peptidoglycan layer.

#### III.D.1. ROS generation and nanorod deformation

Hydroxyl radicals, singlet oxygen, and superoxide anions are known to be produced after ultrafast irradiation of AuNR^8,9,39,40^ Hydroxyl radicals are highly reactive, oxidizing most organic molecules in a near diffusion-limited manner. Superoxide anions can lead to oxidation of Fe-S moieties of dehydratases, chelating and inactivating enzymes which have Fe^2+^ ion co-factors.^41^ Superoxide anions do, however, not readily permeate cell membranes, suggesting a potential synergism between membrane permeation through ultrafast irradiation effects and ROS mediated inactivation.^41^ Singlet oxygen and hydrogen peroxide, which is formed by superoxide, can readily permeate cell membranes. Hydrogen peroxide in the presence of intracellular unincorporated Fe^2+^ can lead to DNA damage through production of hydroxyl radicals *via* Fenton chemistry.^41^

Importantly, the addition of ROS scavengers reverses the reduction of viability observed for AuNR_710 under ultrafast irradiation (Fig. 4), confirming an important role of ROS in the inactivation process. The stronger reduction in viability obtained for AuNR_710 is attributed to more ROS generation than for AuNS and AuNR_750 at these irradiation wavelengths. AuNS are not effective in generating ROS due to a lack of spectral overlap with the excitation laser, while AuNR_750 show a faster degradation than AuNR_710. The specific impacts of different ROS and their corresponding biological pathways towards inactivation are not explored in this study. The temporal and spatial characteristics of ROS generation of free AuNRs under comparable irradiation conditions are discussed in Rajagopal *et al.* 2025.

It is useful to consider the linear estimate of energy per volume absorbed by AuNPs,2
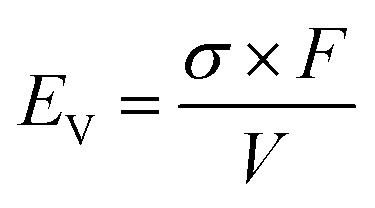
where *F* corresponds to fluence, *V* to volume, and *σ* to absorption cross-section.

FDTD simulations indicate that the absorption cross-section of AuNR_750 is approximately 3 times that of AuNR_710, while AuNR_710 has a volume approximately 20% larger than that of AuNR_750. Hence, AuNR_710 at 9 mJ cm^−2^ has a lower absorbed volumetric energy than AuNR_750 at both 5 and 9 mJ cm^−2^. Excitation of AuNR_710 at 9 mJ cm^−2^ could be optimal for total ROS generation over extended periods of time due to a balance between slower nanorod deformation and slower initial rate of ROS generation than AuNR_750 at 5 or 9 mJ cm^−2^.

Overall, these findings are in agreement with the trends observed for the degradation of Rhodamine B by AuNR mediated ROS generation.^10^ In this prior work, AuNR with plasmon resonances detuned from the ultrafast laser excitation wavelength were also found to be most efficient in ROS generation.

#### III.D.2. Shockwaves and bubbles

In addition to anti-bacterial photothermal and ROS effects, nanocavitation can potentially also occur for the two irradiation conditions considered for both AuNR. The peptidoglycan layer is considered the load-bearing element of the bacterial cell, for Gram-positive bacteria. Indeed, for Gram-positive bacteria, the cell is under a turgor pressure of 20 atm, while that of Gram-negative bacteria is estimated to 0.3–3 atm.^38,42^ Lombard *et al.* simulated pressure shockwaves of 40 MPa for a 50 nm AuNS irradiated with 400 nm 10 ps pulsed irradiation using a fluence of 1.5× the threshold for nanocavitation.^26^ The shockwaves generated through nanocavitation may lead to cell wall damage, perhaps with increased susceptibility in Gram-negative cells. Shockwaves are known to cause transient poration and damage to lipid membranes.^26,43,44^ This could lead to a permeabilization of the cell membrane as indicated by the LIVE/DEAD BacLight (Molecular Probes, Eugene, OR) staining observed in Fig. 4.

#### III.D.3. Influence of external osmotic environment

In this study, we utilized low-salt LB growth media and low-salt buffer (10mM NaCl + 20 mM HEPES), similar conditions to those in studies of metabolism, biofilm production.^45^ Under these hypoosmotic conditions, disruption of the cell wall through our anti-microbial strategy can lead to cell bursting.^46^ Osmotic stress activates general stress responses and can promote cross-tolerance to oxidative and other forms of stress induced by our anti-microbial strategy.^47^ It would be interesting to evaluate the potential synergy of this anti-microbial strategy under hyperosmotic conditions.^48^

### III.E. Post-irradiation induced binding

**Fig. 7:**
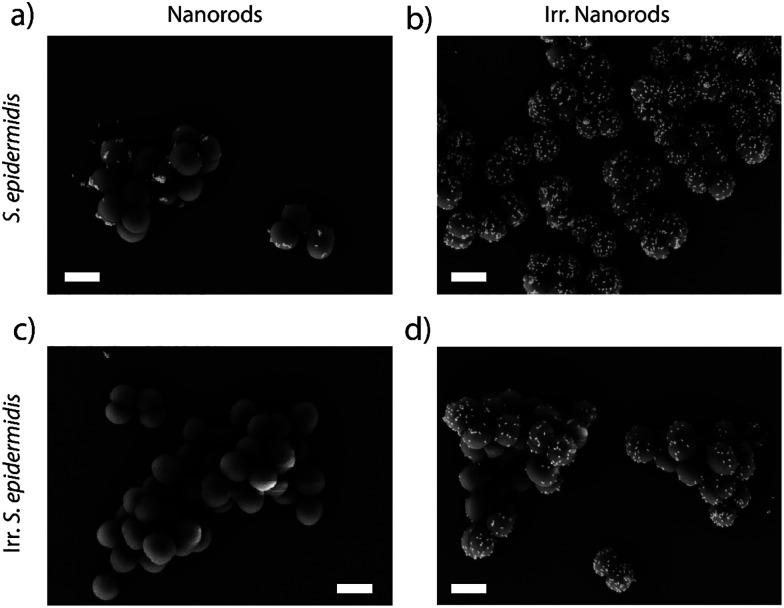
SEM images of AuNP binding *to S. epidermidis* with AuNPs and/or cells irradiated separately for 5 and 30 min respectively under 9 mJ cm^−2^ and then combined. Top row shows binding of (left to right) EPC AuNR_710, followed by irradiated EPC AuNR_710; both with *S. epidermidis.* Bottom row shows binding of EPC AuNR_710 and irradiated EPC AuNR_710, with irradiated *S. epidermidis.* Scalebars denote 1 µm.

Although negatively charged DOPS-coated AuNR do not bind to bacteria, this changes with irradiation as seen in Fig. 2c, d, despite AuNR retaining a negative surface potential. To investigate the nature of this binding interaction, we separately irradiated EPC-coated AuNR_710 and bacteria and then incubated them for 5 min. Nanorods and bacteria were irradiated at 9 mJ cm^−2^ for 5 and 30 min respectively. Irradiation of just nanorods, or of nanorods and bacteria results in efficient binding (Fig. 7). The *ζ* potential of EPC-coated AuNR_710 changed from 28 ± 9 mV to −22 ± 3 mV after irradiation. Binding of nanoparticles to bacteria despite a surface charge of identical sign could be due to counterion-mediated interactions,^49^ long-range same-charge attractive interactions,^50^ or non-electrostatic effects. We note that DOPS-coated AuNR_710 do not exhibit any reduction in viability, hence this post-irradiation induced binding effect does not have an impact on bacterial inactivation. This trend indicates that the DOPS-coated AuNR deform before they bind and is in line with a lack of inactivation seen with AuNS with a positive potential. Finally, DOPS coated AuNS show binding without the need for irradiation, and the substitution of bacteria with polystyrene beads also results in binding, suggesting a shape dependent and surface charge driven process (see Fig. S6 and S7 in SI).

### III.F. Comparison with other methodologies

Nanoparticles and photonic combination therapies are applicable to wound treatment where biofilm formation remains a serious complication.^21,51^ In such a scenario photothermal effects generated by an ensemble of nanoparticles play an important role, potentially leading to tissue damage.^52^ Furthermore, at high input concentrations it becomes challenging to decouple thermal effects under ultrafast irradiation conditions from other potential inactivation strategies,^28^ such as shockwave-mediated activation,^23^ especially if long irradiation times are required as in the case of bacteria. Methodologies, like the one described in this work, that involve the direct binding of nanoparticles to bacteria and that can thus utilize local photothermal and ROS effects provided by the nanoparticles provide opportunities for lowering nanoparticle and light dosages and can thus reduce collateral damage.^53^ The bacterial inactivation observed here for ultrafast irradiation of AuNR is lower than that observed for hybrid systems containing noble metal nanoparticles and photosensitizers or photocatalysts under continuous wave excitation.^17,18^ We attribute this to a limit in total ROS generation associated with the deformation of the AuNR under ultrafast irradiation. Although reshaping limits the antibacterial efficacy of AuNR, it may also provide a self-limiting effect that further minimizes off-target effects. Further optimization of detuning and fluence can improve on the modest bacterial inactivation through ultrafast excitation of AuNR described in this work.

## IV. Conclusion

Ultrafast irradiation of AuNR_710 bound to *S. epidermidis* achieved a significant reduction in bacterial viability, which we attributed to ROS generation with potential additional contributions from a localized photothermal effect and shockwave generation. Unlike irradiation of AuNR_710, irradiation of AuNR_750 failed to achieve a significant reduction in bacterial viability under identical conditions. The increased effect for AuNR_710, whose plasmon resonance has a higher degree of detuning from the excitation wavelength, was attributed to an optimization of the competition between power-dependent structural reshaping of the nanoparticles, and the increase in ROS generation rate for higher fluences. Although the observed bacterial inactivation observed in this work is only modest, our work emphasizes the relevance of detuning of plasmon resonance and excitation wavelength for maintaining ROS regeneration by AuNR under ultrafast excitation. The effect of detuning and AuNR structural reshaping has not been characterized in previous reports of bacterial inactivation studies involving femtosecond irradiation of gold nanorods.^23,28^ It is conceivable that the antibacterial efficacy can be further enhanced in a “sweet spot” of ROS generation and stability. Alternatively, resonant or off-resonant excitation of AuNS,^26,37^ unaffected by deformation and potentially retaining attachment to the bacterial membrane, can be further explored for microbial inactivation.

The plasmonic antibacterial strategy based on ultrafast irradiation of AuNR outlined in this work is applicable under conditions that are compatible with nanoparticle administration and accessible to light irradiation. One example is, for instance, in the treatment of chronic wound infections where biofilms lower the efficacy of antibiotics through a variety of mechanisms, while their often polymicrobial nature requires a broad-spectrum anti-microbials.^21,54,55^ Attempts for bacterial inactivation in this scenario through photothermal effects generated by a nanoparticle ensemble risk additional tissue damage, whereas local ROS generation by nanoparticles bound to bacteria, potentially enhanced by local photothermal effects and shockwave generation, could facilitate a more selective bacterial inactivation using low nanoparticle input and light dosage. This plasmonic antibacterial strategy can furthermore be combined with conventional antibiotics-based strategies. Our live-dead staining experiments indicate that ultrafast irradiation of AuNR_710 bound to *S. epidermidis* induces increased membrane permeability, which provides opportunities to enhance uptake of antibiotics. Furthermore, ROS induced oxidative stress is known to be a crucial mechanism of action for bactericidal drugs.^56,57^ For these classes of drugs, the increase in ROS production is attributed to increased cellular respiration.^57^ This effect can be modulated through mutations which downregulate metabolic processes.^56^ Alternatively, plasmonically enhanced direct production of ROS could be a synergistic tool to combat persisters^58^ after conventional treatment in locations accessible to ultrafast radiation.

## Conflicts of interest

The authors have no conflicts of interest to declare.

## Supplementary Material

TB-014-D5TB02670A-s001

## Data Availability

Data involved in this work is available as a Zenodo repository: https://doi.org/10.5281/zenodo.21267734. Supplementary information (SI): additional information on AuNP characterization, FDTD simulation of AuNR, two-temperature model, SEM sample preparation, LIVE/DEAD assay, and bacteria-AuNP binding. See DOI: https://doi.org/10.1039/d5tb02670a.

## References

[cit1] Ho C. S., Wong C. T. H., Aung T. T., Lakshminarayanan R., Mehta J. S., Rauz S. (2025). *et al.*, Antimicrobial resistance: a concise update. Lancet Microbe.

[cit2] Darby E. M., Trampari E., Siasat P., Gaya M. S., Alav I., Webber M. A. (2023). *et al.*, Molecular mechanisms of antibiotic resistance revisited. Nat. Rev. Microbiol..

[cit3] Miller W. R., Arias C. A. (2024). ESKAPE pathogens: antimicrobial resistance, epidemiology, clinical impact and therapeutics. Nat. Rev. Microbiol..

[cit4] Magnano San Lio R., Favara G., Maugeri A., Barchitta M., Agodi A. (2023). How Antimicrobial Resistance Is Linked to Climate Change: An Overview of Two Intertwined Global Challenges. Int J. Environ. Res. Public Health.

[cit5] An X., Erramilli S., Reinhard B. M. (2021). Plasmonic nano-antimicrobials: Properties, mechanisms and applications in microbe inactivation and sensing. Nanoscale.

[cit6] Chadwick S. J., Salah D., Livesey P. M., Brust M., Volk M. (2016). Singlet oxygen generation by laser irradiation of gold nanoparticles. J. Phys. Chem. C.

[cit7] Gao L., Liu R., Gao F., Wang Y., Jiang X., Gao X. (2014). Plasmon-mediated generation of reactive oxygen species from near-infrared light excited gold nanocages for photodynamic therapy in vitro. ACS Nano.

[cit8] Labouret T., Audibert J. F., Pansu R. B., Palpant B. (2015). Plasmon-Assisted Production of Reactive Oxygen Species by Single Gold Nanorods. Small.

[cit9] Mitiche S., Gueffrache S., Marguet S., Audibert J. F., Pansu R. B., Palpant B. (2022). Coating gold nanorods with silica prevents the generation of reactive oxygen species under laser light irradiation for safe biomedical applications. J. Mater. Chem. B.

[cit10] Rajagopal R., Kundu K., Ouyang T., Nalluri A., Liu G., Ziegler L. D. (2025). *et al.*, Unraveling Plasmon-Enhanced Reactive Oxygen Species Generation through Ultrafast Light. J. Phys. Chem. C.

[cit11] Ko Y. C., Fang H. Y., Chen D. H. (2017). Fabrication of Ag/ZnO/reduced graphene oxide nanocomposite for SERS detection and multiway killing of bacteria. J. Alloys Compd..

[cit12] Xia D., Liu H., Xu B., Wang Y., Liao Y., Huang Y. (2019). *et al.*, Single Ag atom engineered 3D-MnO2 porous hollow microspheres for rapid photothermocatalytic inactivation of *E. coli* under solar light. Appl. Catal., B.

[cit13] Santos G. M., Ferrara F. I., de S., Zhao F., Rodrigues D. F., Shih W. C. (2016). Photothermal inactivation of heat-resistant bacteria on nanoporous gold disk arrays. Opt. Mater. Express.

[cit14] Tatsuno I., Niimi Y., Tomita M., Terashima H., Hasegawa T., Matsumoto T. (2021). Mechanism of transient photothermal inactivation of bacteria using a wavelength-tunable nanosecond pulsed laser. Sci. Rep..

[cit15] Wang Y., Wang Y., Wu J., Kong H., Wu Q., Gong X. (2025). *et al.*, Bacteria-Activated AuNP Plasmonic Coupling:
One-Step Photothermal Platform for Pathogen Detection and Sterilization. Anal. Chem..

[cit16] Kuo W. S., Chang C. N., Chang Y. T., Yeh C. S. (2009). Antimicrobial gold nanorods with dual-modality photodynamic inactivation and hyperthermia. Chem. Commun..

[cit17] An X., Naowarojna N., Liu P., Reinhard B. M. (2020). Hybrid Plasmonic Photoreactors as Visible Light-Mediated Bactericides. ACS Appl. Mater. Interfaces.

[cit18] Ding R., Yu X., Wang P., Zhang J., Zhou Y., Cao X. (2016). *et al.*, Hybrid photosensitizer based on amphiphilic block copolymer stabilized silver nanoparticles for highly efficient photodynamic inactivation of bacteria. RSC Adv..

[cit19] Planas O., Macia N., Agut M., Nonell S., Heyne B. (2016). Distance-Dependent Plasmon-Enhanced Singlet Oxygen Production and Emission for Bacterial Inactivation. J. Am. Chem. Soc..

[cit20] Wang Y., Ding X., Chen Y., Guo M., Zhang Y., Guo X. (2016). *et al.*, Antibiotic-loaded, silver core-embedded mesoporous silica nanovehicles as a synergistic antibacterial agent for the treatment of drug-resistant infections. Biomaterials.

[cit21] Sedighi O., Bednarke B., Sherriff H., Doiron A. L. (2024). Nanoparticle-Based Strategies for Managing Biofilm Infections in Wounds: A Comprehensive Review. ACS Omega.

[cit22] Nazari M., Xi M., Lerch S., Alizadeh M. H., Ettinger C., Akiyama H. (2017). *et al.*, Plasmonic Enhancement of Selective Photonic Virus Inactivation. Sci. Rep..

[cit23] Nazari M., Xi M., Aronson M., Mcrae O., Hong M. K., Gummuluru S. (2019). *et al.*, Plasmon-Enhanced Pan-Microbial Pathogen Inactivation in the Cavitation Regime: Selectivity Without Targeting. ACS Appl. Nano Mater..

[cit24] Lombard J., Biben T., Merabia S. (2014). Kinetics of nanobubble generation around overheated nanoparticles. Phys. Rev. Lett..

[cit25] Lombard J., Biben T., Merabia S. (2015). Nanobubbles around plasmonic nanoparticles: Thermodynamic analysis. Phys. Rev. E: Stat., Nonlinear, Soft Matter Phys..

[cit26] Lombard J., Lam J., Detcheverry F., Biben T., Merabia S. (2021). Strong and fast rising pressure waves emitted by plasmonic vapor nanobubbles. Phys. Rev. Res..

[cit27] Severn M. M., Horswill A. R. (2023). Staphylococcus epidermidis and its dual lifestyle in skin health and infection. Nat. Rev. Microbiol..

[cit28] Jijie R., Dumych T., Chengnan L., Bouckaert J., Turcheniuk K., Hage C. H. (2016). *et al.*, Particle-based photodynamic therapy based on indocyanine green modified plasmonic nanostructures for inactivation of a Crohn's disease-associated Escherichia coli strain. J. Mater. Chem. B.

[cit29] Turcheniuk K., Turcheniuk V., Hage C. H., Dumych T., Bilyy R., Bouckaert J. (2015). *et al.*, Highly effective photodynamic inactivation of E. coli using gold nanorods/SiO2 core–shell nanostructures with embedded verteporfin. Chem. Commun..

[cit30] Ye X., Zheng C., Chen J., Gao Y., Murray C. B. (2013). Using Binary Surfactant Mixtures To Simultaneously Improve the Dimensional
Tunability and Monodispersity in the Seeded Growth of Gold Nanorods. Nano Lett..

[cit31] Ouyang T., Chen Y. C., Kundu K., Zhong X., Mei Y., Nalluri A. (2024). *et al.*, Direct Excitation Transfer in Plasmonic Metal-Chalcopyrite-Hybrids: Insights from Single Particle Line Shape Analysis. ACS Nano.

[cit32] Berger Bioucas F. E., Damm C., Peukert W., Rausch M. H., Koller T. M., Giraudet C. (2019). *et al.*, Translational and Rotational Diffusion Coefficients of Gold Nanorods Dispersed in Mixtures of Water and Glycerol by Polarized Dynamic Light Scattering. J. Phys. Chem. B.

[cit33] Robertson J., McGoverin C., Vanholsbeeck F., Swift S. (2019). Optimisation of the Protocol for the LIVE/DEAD® BacLightTM Bacterial Viability Kit for Rapid Determination of Bacterial Load. Front. Microbiol..

[cit34] Ekici O., Harrison R. K., Durr N. J., Eversole D. S., Lee M., Ben-Yakar A. (2008). Thermal analysis of gold nanorods heated with femtosecond laser pulses. J. Phys. Appl. Phys..

[cit35] Labouret T., Palpant B. (2016). Nonthermal model for ultrafast laser-induced plasma generation around a plasmonic nanorod. Phys. Rev. B.

[cit36] Silva M. G., Teles-Ferreira D. C., Siman L., Chaves C. R., Ladeira L. O., Longhi S. (2018). *et al.*, Universal saturation behavior in the transient optical response of plasmonic structures. Phys. Rev. B.

[cit37] Boulais É., Lachaine R., Meunier M. (2012). Plasma mediated off-resonance plasmonic enhanced ultrafast laser-induced nanocavitation. Nano Lett..

[cit38] Rohde M. (2019). The Gram-Positive Bacterial Cell Wall. Microbiol. Spectr..

[cit39] Guerrero-Florez V., Mendez-Sanchez S. C., Patrón-Soberano O. A., Rodríguez-González V., Blach D., O F. M. (2020). Gold nanoparticle-mediated generation of reactive oxygen species during plasmonic photothermal therapy: a comparative study for different particle sizes, shapes, and surface conjugations. J. Mater. Chem. B.

[cit40] Singh S. K., Mazumder S., Vincy A., Hiremath N., Kumar R., Banerjee I. (2023). *et al.*, Review of Photoresponsive Plasmonic Nanoparticles That Produce Reactive Chemical Species for Photodynamic Therapy of Cancer and Bacterial Infections. ACS Appl. Nano Mater..

[cit41] Imlay J. A. (2013). The molecular mechanisms and physiological consequences of oxidative stress: lessons from a model bacterium. Nat. Rev. Microbiol..

[cit42] Deng Y., Sun M., Shaevitz J. W. (2011). Direct Measurement of Cell Wall Stress Stiffening and Turgor Pressure in Live Bacterial Cells. Phys. Rev. Lett..

[cit43] Kitz M., Preisser S., Wetterwald A., Jaeger M., Thalmann G. N., Frenz M. (2011). Vapor bubble generation around gold nano-particles and its application to damaging of cells. Biomed. Opt. Express.

[cit44] Kodama T., Hamblin M. R., Doukas A. G. (2000). Cytoplasmic Molecular Delivery with Shock Waves:Importance of Impulse. Biophys. J..

[cit45] Pedroza-Dávila U., Uribe-Alvarez C., Morales-García L., Espinoza-Simón E., Méndez-Romero O., Muhlia-Almazán A. (2020). *et al.*, Metabolism, ATP production and biofilm generation by Staphylococcus epidermidis in either respiratory or fermentative conditions. AMB Express.

[cit46] Bremer E., Krämer R. (2019). Responses of Microorganisms to Osmotic Stress. Annu. Rev. Microbiol..

[cit47] Ranganathan N., Johnson R., Edwards A. M. (2020). The general stress response of Staphylococcus aureus promotes tolerance of antibiotics and survival in whole human blood. Microbiology.

[cit48] Connell S., Li J., Shi R. (2013). Synergistic bactericidal activity between hyperosmotic stress and membrane-disrupting nanoemulsions. J. Med. Microbiol..

[cit49] Ise N. (2010). Like likes like: counterion-mediated attraction in macroionic and colloidal interaction. Phys. Chem. Chem. Phys..

[cit50] Wang S., Walker-Gibbons R., Watkins B., Flynn M., Krishnan M. (2024). A charge-dependent long-ranged force drives tailored assembly of matter in solution. Nat. Nanotechnol..

[cit51] Okkeh M., Bloise N., Restivo E., De Vita L., Pallavicini P., Visai L. (2021). Gold Nanoparticles: Can They Be the Next Magic Bullet for Multidrug-Resistant Bacteria?. Nanomaterials.

[cit52] Wang R., Wang X., Mu X., Feng W., Lu Y., Yu W. (2022). *et al.*, Reducing thermal damage to adjacent normal tissue with dual thermo-responsive polymer via thermo-induced phase transition for precise photothermal theranosis. Acta Biomater..

[cit53] Deng X., Shao Z., Zhao Y. (2021). Solutions to the Drawbacks of Photothermal and Photodynamic Cancer Therapy. Adv. Sci..

[cit54] Falanga V., Isseroff R. R., Soulika A. M., Romanelli M., Margolis D., Kapp S. (2022). *et al.*, Chronic wounds. Nat. Rev. Dis. Primers.

[cit55] Han G., Ceilley R. (2017). Chronic Wound Healing: A Review of Current Management and Treatments. Adv. Ther..

[cit56] Dwyer D. J., Collins J. J., Walker G. C. (2015). Unraveling the Physiological Complexities of Antibiotic Lethality. Annu. Rev. Pharmacol. Toxicol..

[cit57] Lobritz M. A., Belenky P., Porter C. B. M., Gutierrez A., Yang J. H., Schwarz E. G. (2015). *et al.*, Antibiotic efficacy is linked to bacterial cellular respiration. Proc. Natl. Acad. Sci. U. S. A..

[cit58] Niu H., Gu J., Zhang Y. (2024). Bacterial persisters: molecular mechanisms and therapeutic development. Signal Transduction Targeted Ther..

