# Supplementary material for: Ultrafast-light driven plasmonic inactivation of S. epidermidis: mechanistic insights
Source: J Mater Chem B. 2026 Jul 13;14(29):9072–82. doi: 10.1039/d5tb02670a (PMC13358809; doi:10.1039/d5tb02670a)
Supplement: TB-014-D5TB02670A-s001 [file TB-014-D5TB02670A-s001.pdf]

Supplementary Information (SI) for Journal of Materials Chemistry B.
This journal is © The Royal Society of Chemistry 2026

Supporting Information: Ultrafast-Light Driven Plasmonic Inactivation of *S. epidermidis* : Mechanistic Insights

Ramprasath Rajagopal,[1,2] Koustav Kundu,[3,2] Saatwik Suman,[3,2] Ainsley Gray,[3,2] Lawrence D. Ziegler,[3,2] Shyamsunder Erramilli,[1,2] and Björn Reinhard[3,2]

[1] *Department of Physics, Boston University*

[2] *Photonics Center, Boston University*

[3] *Department of Chemistry, Boston University*

(Dated: June 11, 2026)

I. TEM AND NANOPARTICLE DISTRIBUTION

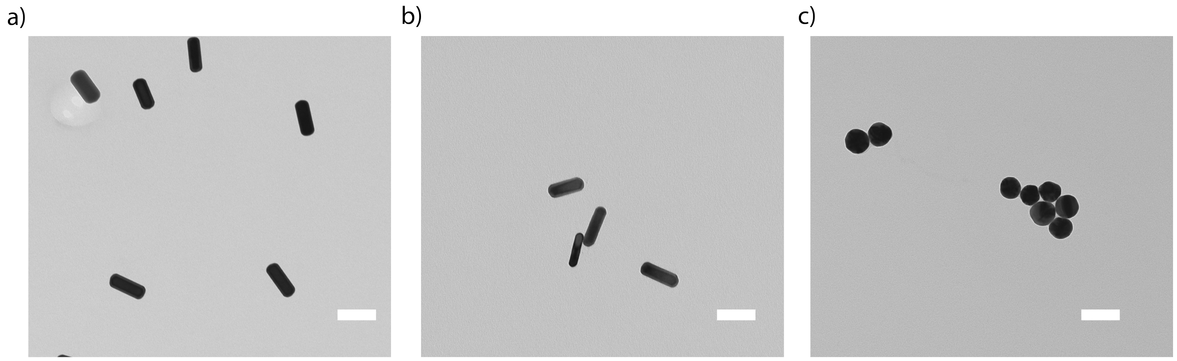

FIG. S1: Representative TEM images of a) AuNR_710 b) AuNR_750, and c) AuNS used in this study. Scalebars are 100 nm

The nanorods were approximated as cylinders with ellipsoidal ends, and AuNS were approximated as spheres. The length and radius of the cylinders along with the axes of the ellipsoidal ends were determined from TEM images. Figures S2 and Figure S3 show the distributions of various physical parameters of the nanoparticles.

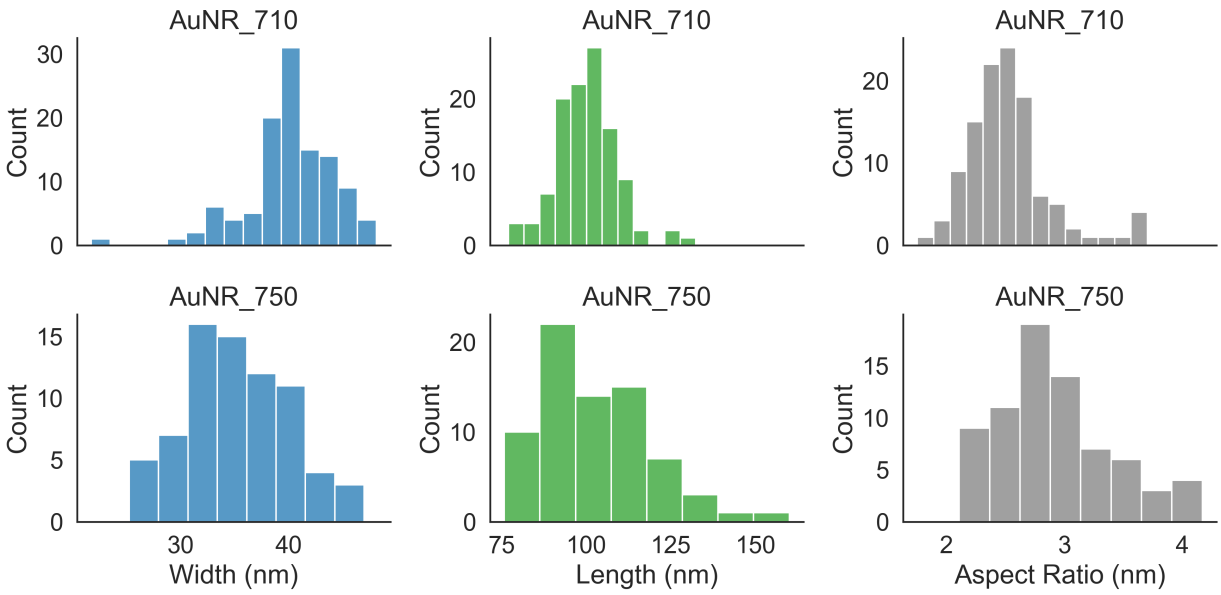

FIG. S2: Width, Length, and Aspect Ratio distributions of AuNR_710 and AuNR_750

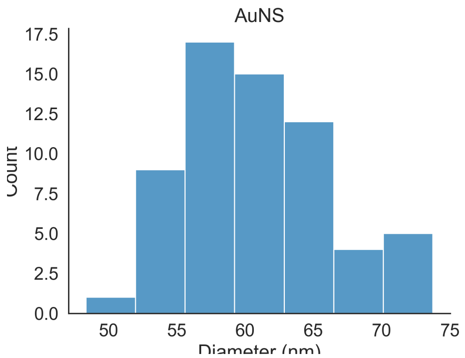

FIG. S3: Diameter distribution of AuNS

II. FDTD SIMULATION

Ansys Lumerical FDTD software [1] was used to simulate the spectrum of different AuNRs. The nanorods were modeled as cylinders with spheroidal ends. A total-field scattered-field (350 nm to 1100 nm) source was used from the x-axis with a polarization angle of 90 º to simulate the extinction spectrum of the AuNRs. Orientation of AuNRs are taken to be parallel to the polarization. The boundary conditions were set as PML in all directions and the background material was considered as water (Palik[2]). For the dielectric function of gold, Johnson and Christy [3] was used . The mesh was set as auto non-uniform and an additional mesh of 0.2 nm in all directions was used for all the simulations. Further mesh refinement did not significantly change cross-sections.

The respective extinction cross-sections and absorption cross-sections of AuNS, AuNR_710, and AuNR_750 are shown in Figure S4

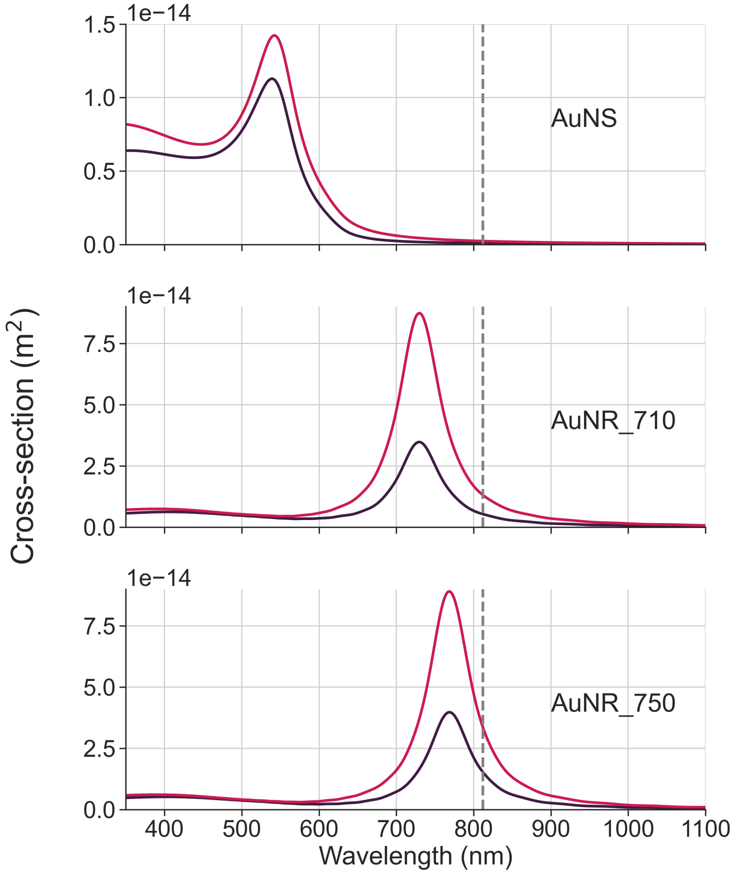

FIG. S4: Extinction cross-sections (red) and absorption cross-sections (black) of AuNS, AuNR_710, and AuNR_750, where AuNRs are aligned to the polarization of E-field. Gray line indicates center wavelength of excitation, 812 nm

III. NANOROD PARAMETERS

	AuNS	AuNR_710	AuNR_750
Length (nm)	-	100 ± 9	103 ± 16
Width (nm)	61 ± 5.5	40 ± 4	36 ± 5
Aspect Ratio	1	2.5 ± 0.4	2.9 ± 0.5
Volume (nm^3)	118,900	116,500	94,360
σ_{abs} @ 812 nm (m^2) (TE)	9.8e-17	5.1e-15	1.5e-14
σ_{sca} @ 812 nm (m^2) (TE)	1.4e-16	7.3e-15	1.8e-14

TABLE S1: Characteristic Parameters of Gold Nanoparticles. Cross-sections of AuNRs were determined when AuNRs longitudinal axis are parallel to the electric field.

The particle concentration is estimated from the slope of OD 400 nm vs. n_{Au} determined in Rajagopal et al. 2025 (Supporting Information Figure S4 b), where 1 O.D. at 400 nm corresponds to 3×10^{-4} mols of Au. The derivation is as follows,

$$n_{\mathrm{AuNP}} = \frac{V \rho_{\mathrm{Au}}}{197.0 \text{ g/mol}}$$

$$\text{Particle conc. at 1O.D.} = \frac{3 \times 10^{-4}\text{mols}}{n_{\mathrm{AuNP}}}$$

where n_{AuNP} refers to number of moles of Au in a certain AuNP. For instance, in bacterial-AuNR_710 sample, the concentration of particles is 2×10^9 particles/mL

IV. THERMAL SIMULATIONS

We adapt a two-temperature model within the gold nanoparticle coupled with heat diffusion in water [4]. We choose the fluence corresponding to a maximum temperature just under 550 K, 1 nm away from the gold nanorod. Maximum lattice temperature of gold nanoparticle reached ≈ 1200 K, with the maximum electronic temperature reaching ≈ 8000 K

$$
\begin{aligned}
\frac{dT_e}{dt} &= g(T_l - T_e) + P_{abs}(t) \\
C_l \frac{dT_l}{dt} &= -g(T_l - T_e) + \frac{\dot{Q}_w}{V_p} \\
Q_w &= A_s G(T_l - T_{w,s}) \\
\rho_w c_w \frac{\partial T_w}{\partial t} &= k \frac{\partial^2 T_w}{\partial t^2}
\end{aligned}
$$

The simulations were conducted using COMSOL multiphysics platform. Thermal conductance, G, is determined from citrus capped AuNP [5]. Figure S5 evaluates the impact of different conductances G. For $10G$ case, the temperature at the 1 nm boundary reaches 650 K (past the bubbling threshold) whereas at 30 nm, the temperature profile is similar to that of G. $0.1G$ exhibits both a lower maximum temperature at 1 nm of 380 K but also a delayed temperature rise 30 nm away while the maximum temperature is about 5 K lower. In summary, the choice of conductance will significantly affect the threshold fluence and lower conductance will exhibit a delayed temperature rise and slightly lower maximum temperature for a given fluence.

From similar thermal simulations performed for AuNR_750, the threshold fluence is estimated to be $\approx 1.5mJ/cm^2$. The temperature contour of AuNR_750 and the temperature vs. time plots are shown in Figure S6.

**Gold (bulk) properties**	
The electron heat capacity, C_e (J/m^{3}K)	from Lin et al. (2008)[6]
Specific heat of lattice, C_l (J/m^{3}K)	$2.49 \cdot 10^6$
Electron-lattice coupling factor, g (W/m^{3}K)	from Lin et al. (2008)[6]
Density, ρ_{gold} (kg/m^3)	19,300
Thermal conductivity, k_{gold} (W/mK)	320
Melting temperature, T_m (K)	1337
**Water properties at standard conditions**	
Density, ρ_w (kg/m^3)	1000
Specific heat, c_{pw} (kJ/kgK)	4.184
Thermal conductivity, k_w (W/mK)	0.61
**At the gold/water interface**	
Thermal conductance, G (W/m^{2}K)	$105.0 \cdot 10^6$ [5]

TABLE S2: Properties of gold and water, and thermal conductance at the gold/water interface.

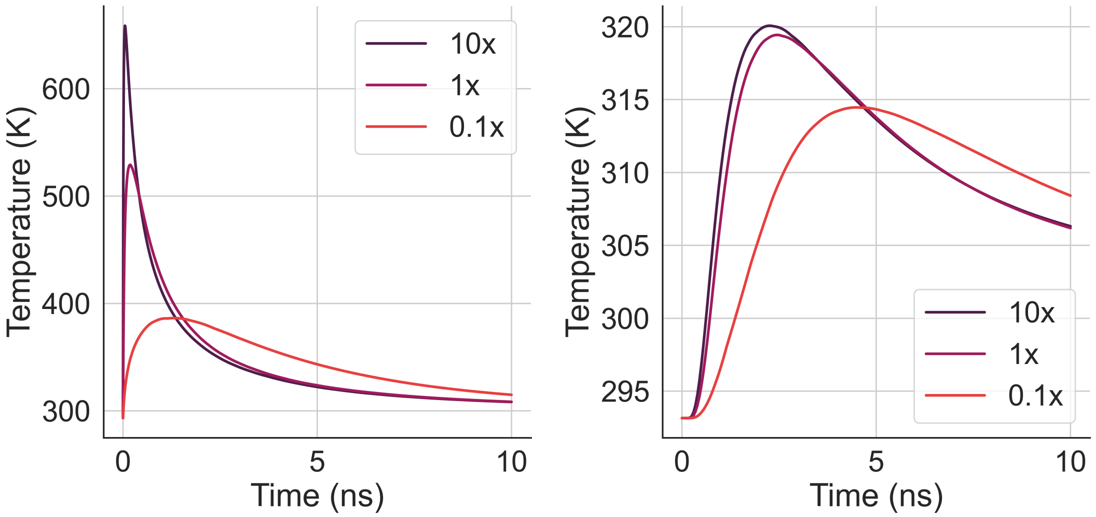

FIG. S5: Effect of variation of thermal conductance, G at gold-water interface at 1 nm (left) and 30 nm (right) for AuNR_710 at the threshold fluence. Legend denotes the scaling of G

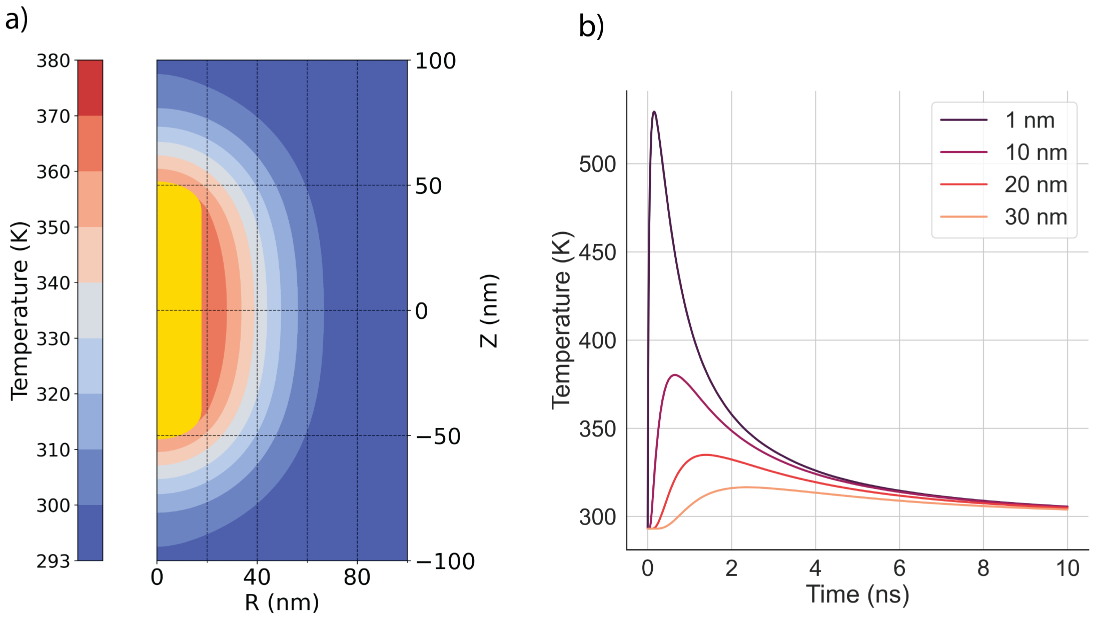

FIG. S6: a) Contour plot of temperature of water surrounding AuNR_750, 2 ns after irradiation with a pulse energy just below the bubbling threshold. b) Corresponding temperature (K) vs. time at various radial distances from the waist of the gold AuNR (Z = 0). 1 nm corresponds to the typical length used for determination of the photothermal bubbling threshold.

V. ADDITIONAL DARK CONTROLS FOR BACTERIA-NANOPARTICLE PLATE ASSAYS

To ascertain that residual CTAB (12 nM), EPC (13 nM), and ROS Scavengers (as detailed in main manuscript) do not affect bacterial viablity, plate counts after incubation for 30 mins were divided by plate counts of bacteria in buffer. For CTAB and EPC, the upper limit of residual concentrations was determined assuming that AuNR_710, at 4×10^9 particles/mL, were exclusively coated with EPC or CTAB and subsequently completely detached and released into the bulk. The area per molecule at the AuNR surface for EPC was taken to be 0.7 nm^2 and that of CTAB was 0.8 nm^2.

For AuNR_710 (EPC, DOPS) we performed dark controls in a similar fashion as bacterial-nanoparticle experiments in the main manuscript: incubating for 5 mins under stirring in a quartz cuvette, subsequently plating, and then leaving for additional 30 minutes followed

by plating.

Results are shown in Figure S7.

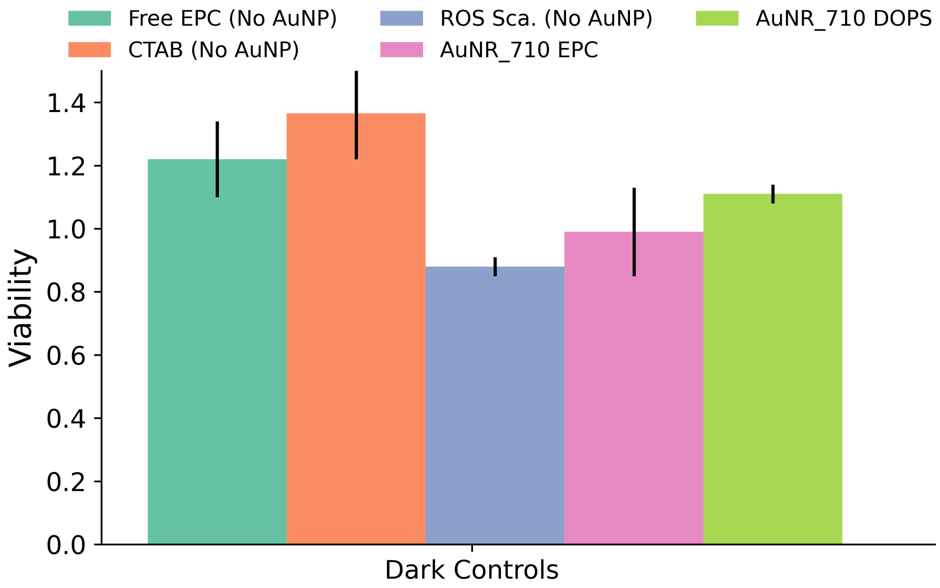

FIG. S7: Controls performed in Dark. Free EPC, CTAB, ROS Scavenger samples do not have any AuNP and are compared with bacteria in buffer whereas the dark AuNR_710 EPC and AuNR_710 DOPS controls follow the same procedure in the main manuscript.

VI. LIVE/DEAD ANALYSIS

LIVE/DEAD® BacLightTM Bacterial Viability Kit (BacLight Kit) was used and the manufacturer's protocol was adapted for fluorescence microscopy. Glass cover slips were soaked in 1 M NaOH solution, and subsequently rinsed to form a hydrophilic layer. 20 μL of bacterial solution were deposited onto slide along with 20 μL of LIVE/DEAD solution and left for about 15 mins. Subsequently excess fluid was aspirated and rinsed with DI water. Subsequently, they were imaged in an inverted microscope. Presence of gold nanoparticles did not noticeably affect results, and care was taken to minimize the time between finishing exposures of samples to being imaged under the fluorescence microscope.

Fluorescence images were processed through wavelet based background filtering technique [7]. Then, a triangle thresholding algorithm is used to segment the clusters in red and green channels separately. This is a parameter-less global threshold algorithm, and as seen in Figure S8, captures most of the clusters present. We note that Figure S8 is significantly

gamma adjusted. This choice leads to conservative segmentation; a more sophisticated algorithm could capture the correct extent of clusters and also detect dim clusters. After that, a mask was constructed based on the union of two channels. The fluorescence ratio was computed per cluster and statistics were computed.

Figure S9 and S10 show LIVE/DEAD controls and the minimal effect of AuNR_710 addition on the fluorescence ratio respectively for live bacteria.

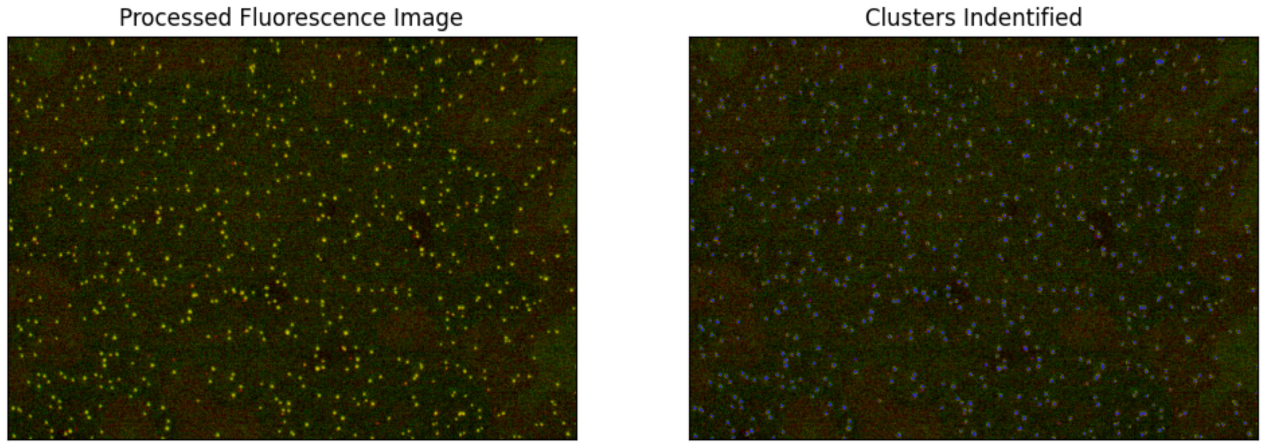

FIG. S8: Image after background subtraction (left) and clusters identified overlayed in blue (right). Images are gamma adjusted (gamma = 0.2)

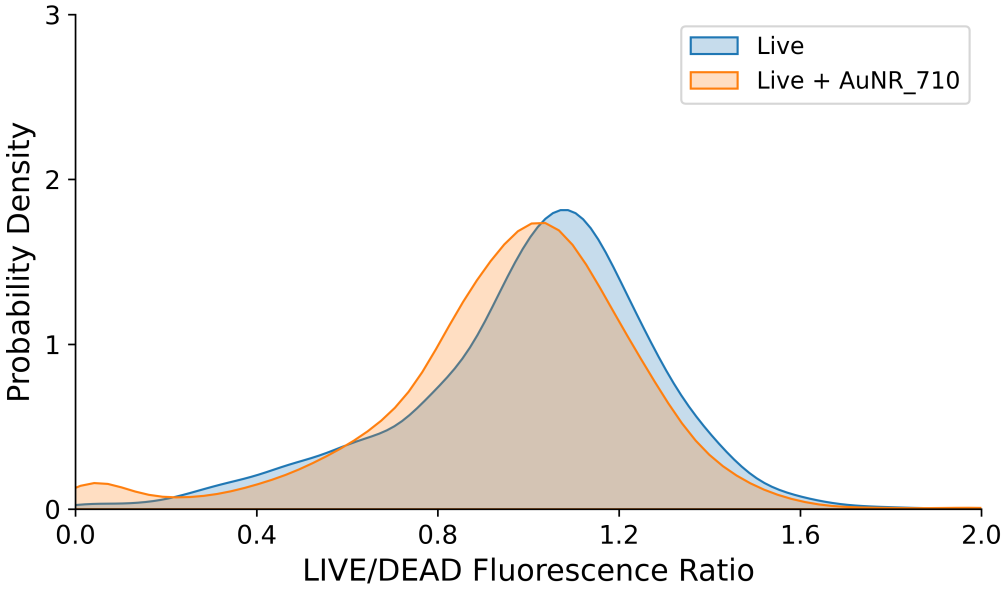

FIG. S10: Probability density estimated from cluster statistics of live bacteria with AuNR_710 and live bacteria without any AuNPs. There is a small peak shift

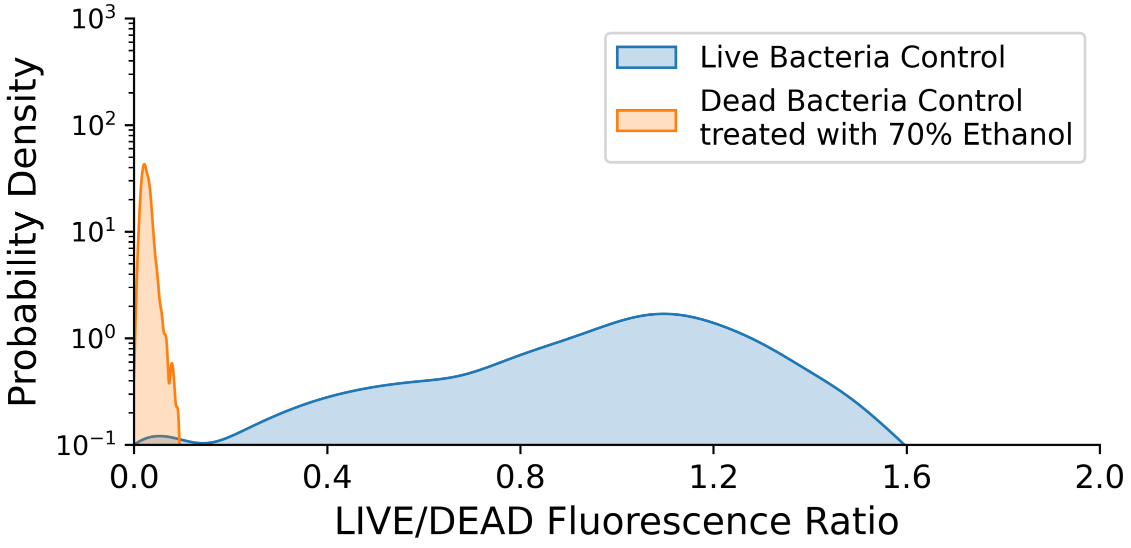

FIG. S9: Probability density estimated from cluster statistics of live bacteria control and dead bacteria control prepared according to manufacturer's protocol for LIVE/DEAD staining. Probability density is ploted on a log-scale to visualize the disparate distributions

VII. SEM SAMPLE PREPATION FOR BACTERIA AND BACTERIA-NANOPARTICLE MIXTURES

Bacterial samples or Bacteria with nanoparticles samples were deposited on a Si wafer within wells of 12-well plate. After 30 mins, a 3% glutaraldehyde solution was gently deposited into wells and left for about an hour. The wells were then rinsed with DI water followed by ethanol dehydration, and gold sputtering.

VIII. ADDITIONAL BINDING TESTS

Separating unbound NPs by centrifugation was challenging and heteroaggregation of bacteria and nanoparticles was difficult to avoid. As an alternative, filtration with a 0.22 um filter was attempted to separate the unbound NPs, followed by quantification with MP-AES. In that case, though, we found that the concentration of NPs studied in the binding experiment was below the detection limit of our instrument, which prevented direct quantification of the bound/unbound fraction of NPs by MP-AES.

We conducted time-series UV-vis spectroscopy investigation to quantify the binding of

AuNP to bacteria. The aggregation that occurs when nanoparticles bind to bacteria is clearly indicated in Figure S11, where AuNRs with EPC display significant broadening and red-shift whereas AuNRs with DOPS do not. AuNS display a similar behavior, AuNS DOPS is found to bind to bacteria while being well separated as indicated by SEM images. To rule out aggregation of the particles in the bulk, the same set of experiments were conducted without bacteria, as seen in Figure S12 and Figure S13. As expected, no significant changes are observed in the UV-Vis spectra.

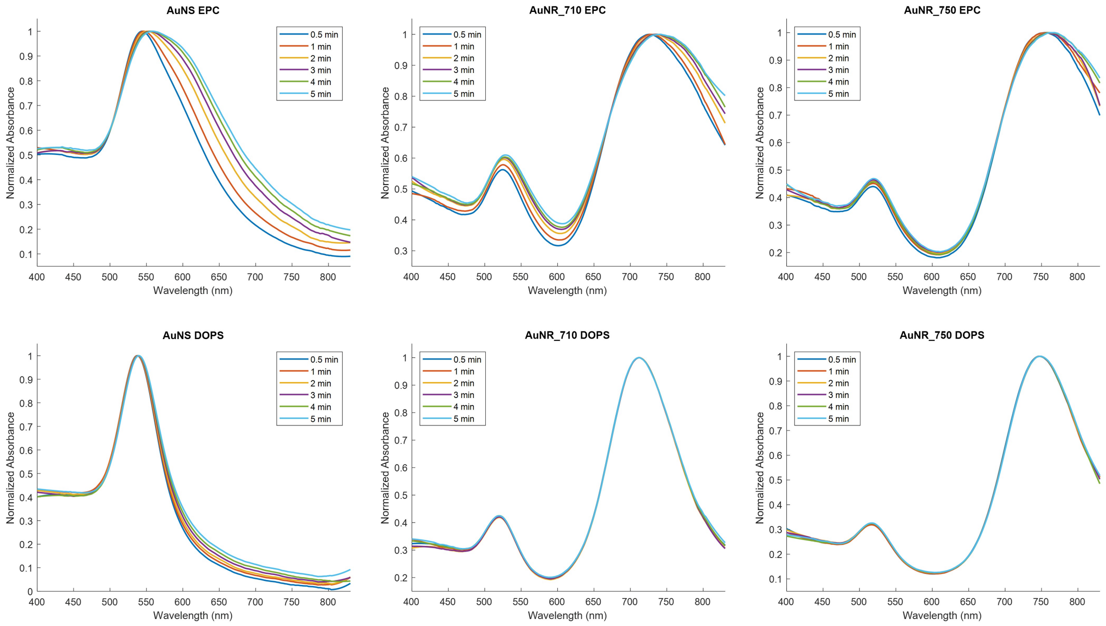

FIG. S11: Time-series of UV-vis spectra of AuNP with EPC or DOPS after mixing with bacteria. For AuNR_710 and AuNR_750 with DOPS, there is no significant change whereas for AuNR_710, AuNR_750 with EPC, there is a gradual red-shift and broadening attributed to binding and subsequent nanoparticle aggregation on bacteria

Finally, to confirm that the binding displayed is charge driven, we tested binding of bacteria incubated with DOPS AuNS (Figure S14), with a ζ potential of -27 ± 3 mV. We also replaced the bacteria with polystyrene beads, retaining a negative surface potential, and still observed binding (Figure S15).

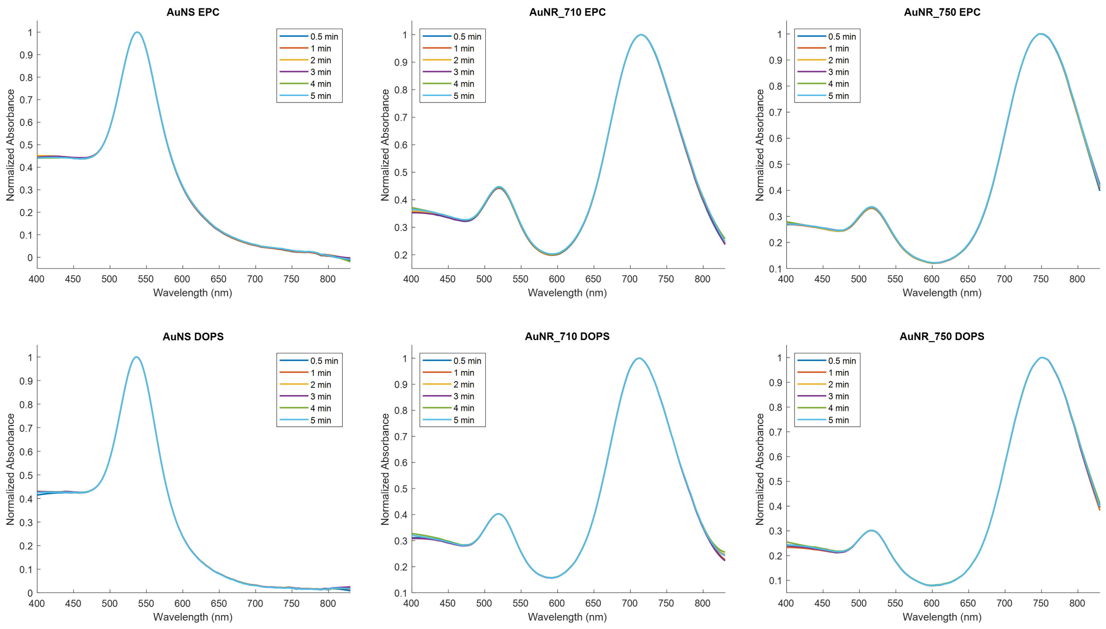

FIG. S12: Time-series of UV-vis spectra of AuNP with EPC or DOPS when no bacteria is present. There are insignificant changes.

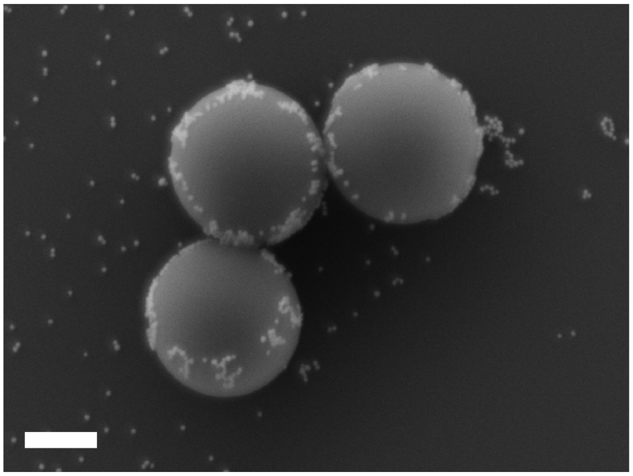

FIG. S15: Polystyrene beads, as a control with a negative surface potential, shows binding with DOPS AuNS. Scalebar is 500 nm

[1] A. Lumerical, FDTD.

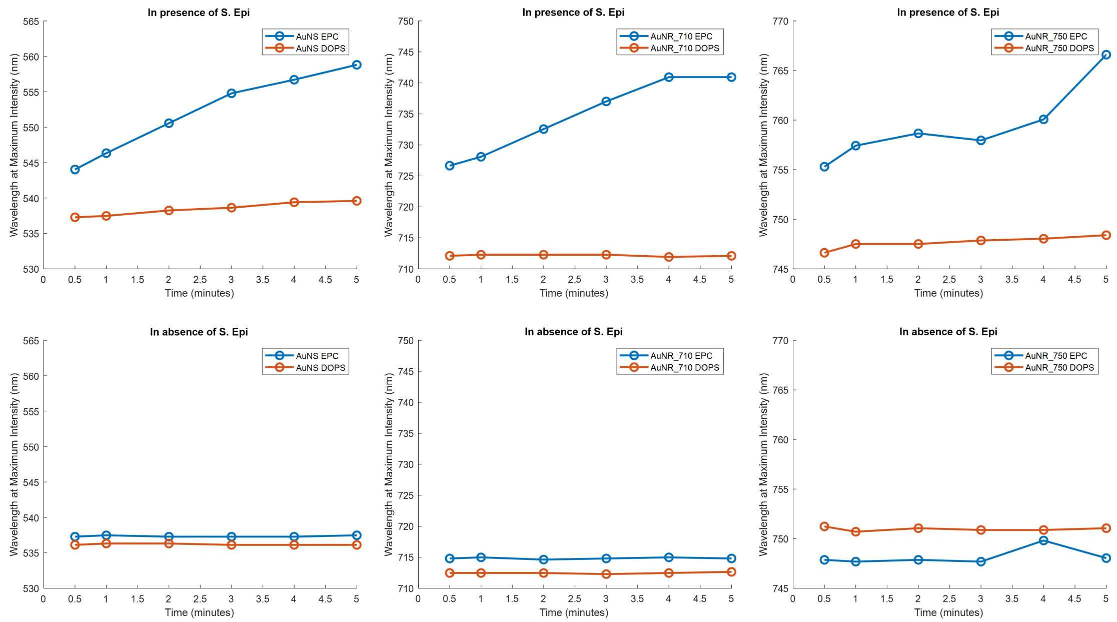

FIG. S13: Peak Wavelength over time (5 mins) of AuNP with EPC (blue) or DOPS (orange), and with and without bacteria

[2] M. R. QUERRY, D. M. WIELICZKA, and D. J. SEGELSTEIN, Water (h2o), in *Handbook of Optical Constants of Solids*, edited by E. D. PALIK (Academic Press, Boston, 1998) pp. 1059–1077.

[3] P. B. Johnson and R. W. Christy, Optical Constants of the Noble Metals, Physical Review B **6**, 4370 (1972), publisher: American Physical Society.

[4] O. Ekici, R. K. Harrison, N. J. Durr, D. S. Eversole, M. Lee, and A. Ben-Yakar, Thermal analysis of gold nanorods heated with femtosecond laser pulses, Journal of Physics D: Applied Physics **41**, 10.1088/0022-3727/41/18/185501 (2008).

[5] A. Plech, V. Kotaidis, S. Grésillon, C. Dahmen, and G. von Plessen, Laser-induced heating and melting of gold nanoparticles studied by time-resolved x-ray scattering, Physical Review B **70**, 195423 (2004), publisher: American Physical Society.

[6] Z. Lin, L. V. Zhigilei, and V. Celli, Electron-phonon coupling and electron heat capacity of metals under conditions of strong electron-phonon nonequilibrium, Physical Review B **77**, 075133 (2008), publisher: American Physical Society.

[7] M. Hüpfel, A. Y. Kobitski, W. Zhang, and G. U. Nienhaus, ENWavelet-based background and noise subtraction for fluorescence microscopy images, Biomedical Optics Express **12**, 969

(2021), publisher: Optica Publishing Group.

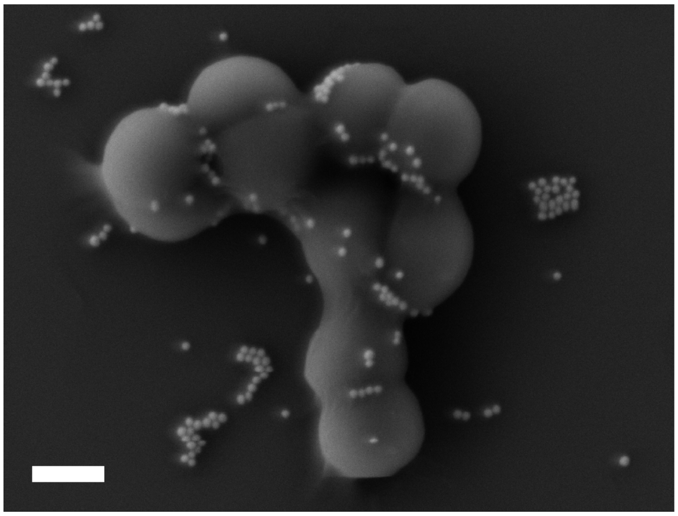

FIG. S14: *S. epidermidis* with DOPS AuNS, binding is observed despite the negative surface potential of both bacteria and nanoparticles. Scalebar is 500 nm
